# Idiopathic right common iliac artery aneurysm in a three-year-old child - case report

**DOI:** 10.1590/1677-5449.200195

**Published:** 2021-06-11

**Authors:** Loraine Entringer Falqueto, Gabriela Caetano Lopes Martins, Arthur Akio Konno Saito, Ayrton Alves Aranha, Ziliane Caetano Lopes Martins

**Affiliations:** 1 Hospital Pequeno Príncipe – HPP, Curitiba, PR, Brasil.; 2 Universidade Federal do Paraná – UFPR, Curitiba, PR, Brasil.

**Keywords:** congenital aneurysm, iliac aneurysm, pediatrics, child

## Abstract

The incidence of iliac aneurysms in children is unknown and there are only a small number of case reports in the literature on the subject. This article describes the case of a 3-year-old male patient with an isolated saccular aneurysm at the bifurcation of the right common iliac artery, of idiopathic origin, which was repaired by resection, ligature of the internal iliac artery and end-to-end vascular anastomosis. After 1 month of follow-up, he was diagnosed with asymptomatic occlusion of the anastomosis. The occlusion had no clinical repercussions because of collateral circulation and the child has had a favorable clinical course over the medium term.

## INTRODUCTION

The incidence of iliac aneurysms in children is unknown and there are only a small number of case reports on the subject in the literature.^1^ The most common etiologies of arterial aneurysms include infection, arterites, connective tissue diseases, fibromuscular dysplasia, and trauma, and idiopathic aneurysms are the least frequent.^2^

When diagnosed, iliac artery aneurysms should be treated immediately, because of the high risk of rupture, thrombosis, or distal embolization.^3^ Treatment is challenging in the pediatric population because of the smaller size of anatomic structures, the patient’s growth, and the long life expectancy.^4^

This article describes the case of a three-year-old male patient with an isolated aneurysm of the right common iliac artery of idiopathic origin. It covers clinical work-up, treatment, and the medium-term results of the case, with the aim of contributing to improvements in treatment of aneurysms of major vessels in children.

## CASE DESCRIPTION

This report was approved by the Research Ethics Committee (ruling number 4.306.931) and the patient’s guardians gave their consent to publication. The patient was male, aged 3 years and 7 months, weighed 17 kg, had brown skin, and a history of recurrent pharyngotonsillitis. He had presented at an Urgent Care Center with fever (axillary temperature of 39.5 °C), myalgia, odynophagia, headaches, vomiting, and diarrhea around 9 days previously. He had no history of prior surgery, comorbidities, or allergies. Abdominal physical examination found a palpable pulsating mass of around 6 cm was in the right iliac fossa. The patient had no family history of aneurysmal disease.

**Figure 1:**
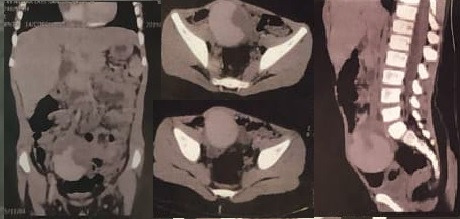
Coronal, axial, and sagittal tomographic images with contrast showing the aneurysm of the right common iliac artery.

Computed tomography (CT) with contrast of the abdomen and pelvis showed an expansive, saccular formation at the bifurcation of the right common iliac artery, measuring 6.5 x 5.6 x 4.8 cm (Figure 1). In view of this diagnosis, the patient was referred as an emergency case to a specialist pediatric vascular surgery center.

At admission, the patient was hemodynamically stable, with no right lower limb skin changes, free from edema, and strength, musculature, and peripheral pulses were all symmetrical with the contralateral limb. No additional findings were recorded beyond the palpable pulsating abdominal mass. He was assessed by the rheumatology team to investigate inflammatory or autoimmune etiologies, and by the infectiology team to rule out a hypothesis of mycotic aneurysm. No additional genetic investigation was conducted because of the absence of family history or suspicious phenotypical abnormalities.

Supplementary examinations conducted included transthoracic echocardiogram, which was normal, abdominal ultrasonography (USG), which showed absence of the left kidney and aneurysmal dilatation of the right iliac artery (4.4 x 4.2 cm) with an associated mural thrombus and a 3.1 cm thrombus-free lumen, and CT of the head which was normal. Cultures of urine, blood, and oropharyngeal swab samples were all negative. General laboratory tests were ordered and the results were all normal except for erythrocyte sedimentation rate (ESR), at 108 mm/h, and C-reactive protein (CRP), at 29.4 mg/L, both of which are elevated levels. On the basis of these results, assays to test for vasculitis were ordered, including immunoglobulins (IgA and IgG were elevated and IgM was normal: 572, 1,308, and 90 mg/dL, respectively) and antineutrophil cytoplasmic antibodies (ANCA), which was negative. Serum tests for hepatitis B and C, tuberculin testing with purified protein derivative (PPD), and the anti-HIV test were all negative.

Despite the elevated inflammatory and total immunoglobulin results, it was not possible to determine an etiology (autoimmune or infectious). In an attempt to reduce the systemic inflammatory response and improve the surgical results, the pediatric rheumatology team prescribed a therapeutic test with methylprednisolone (30 mg/kg/day) for 5 days. Prior to the pulse therapy, prophylaxis was administered with albendazol. The tests were repeated after the corticoid course and there were improvements in ESR (32 mm/h) and CRP (< 5 mg/L). In order to contribute to a diagnosis by biopsy and culture of the aneurysm and to avert complications such as rupture, thromboembolism, and dissection, surgical treatment was initiated.

**Figure 2:**
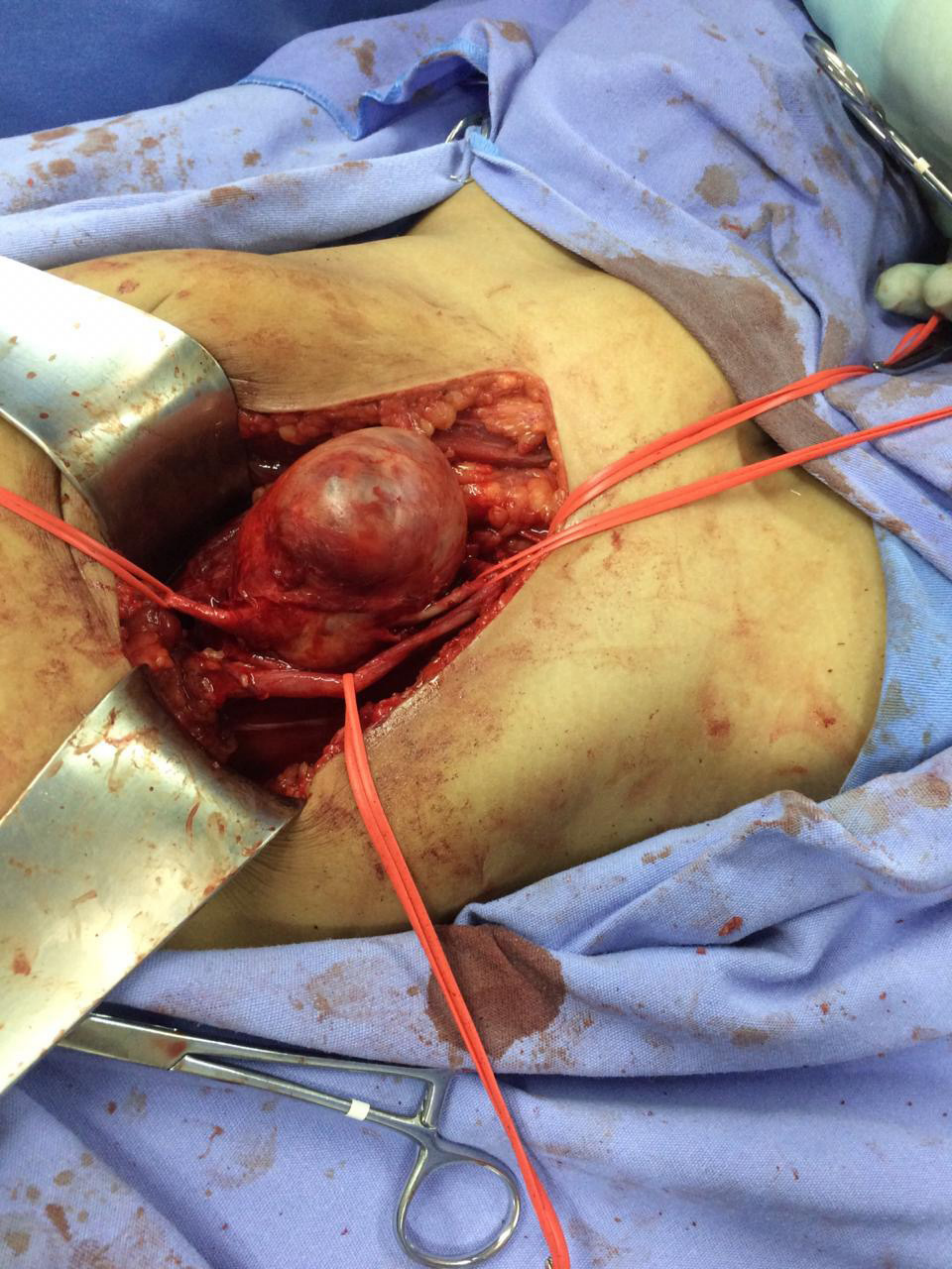
Intraoperative photograph of the idiopathic isolated right common iliac artery aneurysm, with proximal and distal control.

**Figure 3:**
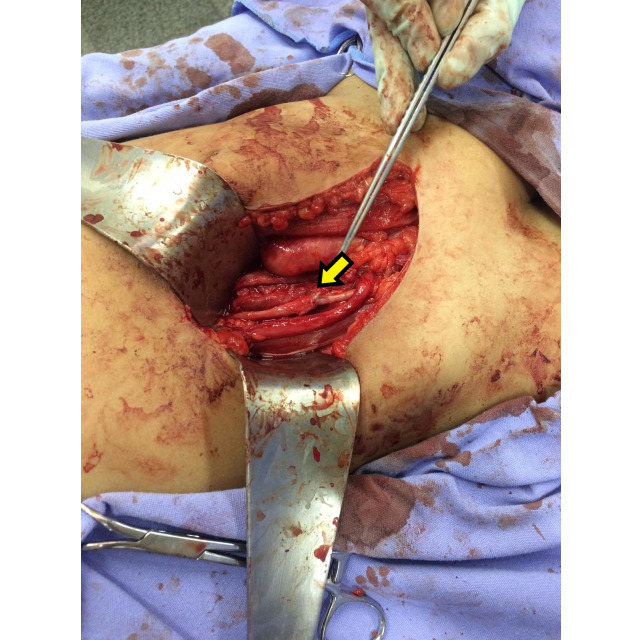
Intraoperative photograph showing the final appearance of the end-to-end anastomosis of the common iliac right external iliac (arrow) arteries after resection of the aneurysm.

The surgical approach chosen was a J-shaped incision in the right iliac fossa and dissection of the extraperitoneal space (a Gibson incision). After proximal repair of the common iliac artery and distal repair of the external iliac artery (Figure 2), the saccular aneurysm was opened and the ostium of the internal iliac artery was hemostatically sutured. The thrombi were removed and the aneurysmal segment was resected. Vascular reconstruction was performed with an end-to-end anastomosis between the common iliac artery and the external iliac artery, with separate 6.0 polypropylene sutures (Figure 3) and the aid of a surgical loupe at 2.5 times magnification. The procedure lasted 4 hours, with no intercurrent conditions and without a need for blood transfusion. The anatomopathological report of analysis of the aneurysm wall and thrombi described fibrous mural and intimal thickening with focal calcified atheroma and recent thrombi in the process of organization. The culture was negative. The aneurysm was classified as congenital/idiopathic.

The patient was kept under observation in the intensive care unit for 48 hours. He remained in hospital for the first 6 postoperative days, on 10 mg/day enoxaparin. Daily physical examinations found symmetrical, well-perfused lower limbs with symmetrical peripheral pulses. Doppler USG showed that the anastomosis was patent. After hospital discharge, the patient was kept on acetylsalicylic acid at 50 mg per day.

At a 1-month outpatients follow-up consultation, the right lower limb was free from edema and skin changes, but the peripheral pulse was not palpable. Control Doppler USG showed occlusion at the level of the anastomosis, but with collateral circulation and without clinical repercussions. Over 13 months of conservative follow-up, no differences in muscle growth were observed between the two lower limbs.

## DISCUSSION

Aneurysms are rare pathologies in pediatrics, particularly isolated aneurysms involving large vessels.^5^ Sarkar et al.^2^ classified arterial aneurysms in children into nine types: I) caused by arterial infection; II) giant-cell aortoarteritis; III) autoimmune connective tissue disease; IV) Kawasaki disease; V) involving degeneration of the tunica media (Ehlers-Danlos syndrome or Marfan's syndrome); VI) associated with other forms of noninflammatory medial degeneration; VII) associated with arterial dysplasia; VIII) congenital/idiopathic aneurysms; and IX) pseudoaneurysms caused by vascular injury.

Congenital/idiopathic aneurysms are uncommon and demand detailed investigation to rule out other causes.^3^ In the case described here, the aneurysm was classified as congenital/idiopathic because of an absence of clinical, laboratory, or pathology findings indicative of other etiologies such as infection, trauma, vascular malformation, or connective tissue diseases.

Based on the patient’s prior history of recurrent tonsillitis, the primary hypothesis was a mycotic aneurysm. However, this was not confirmed by the biopsy and the patient had no further infections after receiving appropriate treatment. Auto-antibody tests are useful for preoperative diagnosis of autoimmune vasculitis. During the physical examination, assessment of stature, joint hypermobility, skin, and facial and limb characteristics are essential elements in identifying or ruling out of genetic and autoimmune abnormalities, guiding investigation. Previous pathologies, family history, and a complete physical examination are crucial to arrive at the correct diagnosis. Histological findings of intimal fibroplasia are common in idiopathic aneurysms and are probably secondary to the process rather than an etiologic factor.^2^

Surgical treatment is the standard option because of the natural history involving progression to rupture, thrombosis, and thromboembolism.^2^^,^ 3 There are reports in the literature describing use of grafts (prosthetic grafts, cryopreserved veins, and autologous grafts), embolization, plication, and resection with primary anastomosis.^1^^,^ 3 ^,^ 6 ^-^ 9 The last of these was chosen in this case. It is recommended that single sutures be used all around the anastomosis circumference, since continuous sutures can be a risk factor for stenosis as the child grows.^6^^,^ 10 There is also the endovascular approach, which is emerging as a promising option for pediatric vascular repair.^11^

Patients with idiopathic aneurysms should be monitored because of the risk of relapse, new aneurysms, or complications related to repair. Doppler USG is the imaging exam of choice.^10^ Davis et al.^12^ recommend annual follow-up. Another recommendation is to use an antiplatelet drug for 6 months after intervention.

In this patient, platelet antiaggregation was maintained and occlusion at the level of the anastomosis was identified at 1 month follow-up, with no clinical repercussions. This was managed conservatively and no growth discrepancies between the limbs were observed up to the most recent consultation, at 13 months’ follow-up. Nevertheless, long-term follow-up will be needed to rule out sequelae related to development of the child and the limb.

It is concluded that management of iliac artery aneurysms in children is challenging both because of the rarity of cases and because of anatomic limitations (dimensions) and the child’s growth. Surgical management is the first choice, but there is no consensus on the exact moment to intervene or on the preferred surgical technique. In the case described, resection and primary anastomosis were chosen. Despite occlusion of the anastomosis at 1 month, treatment of the aneurysm was successful. To date, no sequelae have been observed that affect the patient’s development, although long-term follow-up is essential.
