# Supplementary material for: Idiopathic right common iliac artery aneurysm in a three-year-old child - case report
Source: J Vasc Bras. 2021 Jun 11;20:e20200195. doi: 10.1590/1677-5449.200195 (PMC8210647; doi:10.1590/1677-5449.200195)
Supplement: Translation

# Translation: Aneurisma idiopático de artéria ilíaca comum direita em criança de três anos - relato de caso

**Authors:** Loraine Entringer Falqueto, Gabriela Caetano Lopes Martins, Arthur Akio Konno Saito, Ayrton Alves Aranha, Ziliane Caetano Lopes Martins

**Affiliations:** 1 Hospital Pequeno Príncipe – HPP, Curitiba, PR, Brasil.; 2 Universidade Federal do Paraná – UFPR, Curitiba, PR, Brasil.

**Keywords:** aneurisma congênito, aneurisma ilíaco, pediatria, criança

## Abstract

A incidência de aneurismas ilíacos em crianças não é conhecida, havendo apenas alguns relatos de casos na literatura sobre o tema. Neste estudo, é relatado o caso de um paciente masculino, 3 anos, com aneurisma sacular isolado na bifurcação da artéria ilíaca comum direita de origem idiopática, que foi submetido a ressecção, ligadura da artéria ilíaca interna e anastomose vascular término-terminal. Após 1 mês de seguimento, foi diagnosticada oclusão assintomática da anastomose. Devido à presença de circulação colateral, não houve repercussões clínicas da oclusão, e a criança apresentou evolução clínica favorável a médio prazo.

## INTRODUÇÃO

A incidência de aneurismas ilíacos em crianças não é conhecida, havendo apenas alguns relatos de casos na literatura sobre o tema^1^. As etiologias mais frequentes de aneurismas arteriais incluem infecção, arterites, doenças do tecido conjuntivo, displasia fibromuscular e trauma, sendo os aneurismas idiopáticos os menos frequentes^2^.

Quando diagnosticados, os aneurismas de artéria ilíaca devem ser prontamente abordados devido ao alto risco de rotura, trombose ou embolização distal^3^. O tamanho reduzido das estruturas anatômicas na população pediátrica, o crescimento do paciente e a longa expectativa de vida tornam o tratamento desafiador^4^.

Este artigo aborda um paciente masculino de 3 anos de idade com aneurisma isolado de artéria ilíaca comum direita de origem idiopática. Serão detalhados a investigação clínica, o tratamento e a evolução do caso a médio prazo a fim de contribuir para os avanços no tratamento de aneurismas de grandes vasos em crianças.

## DESCRIÇÃO DO CASO

Este relato foi aprovado pelo Comitê de Ética em Pesquisa (número do parecer 4.306.931), e os responsáveis pelo paciente consentiram com a sua realização. O paciente era do sexo masculino, pardo, 3 anos e 7 meses, 17 kg, com antecedente de faringoamigdalite de repetição. Havia sido atendido em Pronto Atendimento com febre (temperatura axilar de 39,5 °C), mialgia, odinofagia, cefaleia, vômitos e diarreia há cerca de 9 dias. Ele não possuía histórico de cirurgias prévias, comorbidades ou alergias. Ao exame físico abdominal, foi palpada massa pulsátil com cerca de 6 cm em fossa ilíaca direita. Além disso, o paciente também não tinha antecedente familiar de doença aneurismática.

Foi realizada tomografia computadorizada (TC) de abdome e pelve com contraste, a qual evidenciou uma formação expansiva sacular na bifurcação da artéria ilíaca comum direita de 6,5 x 5,6 x 4,8 cm (Figura 1). Devido ao diagnóstico, o paciente foi encaminhado com urgência a um centro de referência pediátrico com cirurgia vascular.

**Figura 1:** Imagens tomográficas com contraste do aneurisma de artéria ilíaca comum direita – coronal, axial e sagital.

Na admissão, o paciente encontrava-se estável hemodinamicamente, com membro inferior direito sem lesões cutâneas, sem edema e com força, trofismo e pulsos periféricos simétricos ao contralateral. Não foram registrados outros achados além da massa abdominal palpável pulsátil. Ele foi avaliado pela reumatologia, para investigar etiologias inflamatórias ou autoimunes, e pela infectologia, sob hipótese de aneurisma micótico. Não foi realizada investigação genética adicional pela ausência de história familiar e alterações fenotípicas suspeitas.

Entre os exames complementares realizados, o ecocardiograma transtorácico estava normal. A ultrassonografia (USG) de abdome demonstrou ausência do rim esquerdo e dilatação aneurismática da artéria ilíaca direita (4,4 x 4,2 cm), com trombo mural associado e 3,1 cm de luz não trombosada. A TC de crânio estava normal. As culturas de urina, sangue e *swab* orofaríngeo foram negativas. Foram solicitados exames laboratoriais gerais, que estavam normais, exceto pela velocidade de hemossedimentação (VHS) — 108 mm/h — e pela proteína C reativa (PCR) — 29,4 mg/L —, as quais estavam aumentadas. Com base nessas alterações, para investigação de vasculites, foram dosados imunoglobulinas (IgA e IgG aumentados e IgM normal: 572, 1.308 e 90 mg/dL, respectivamente) e anticorpo anticitoplasmático de neutrófilo (ANCA), o qual foi negativo. As sorologias para hepatite B e C, o teste tuberculínico com derivado proteico purificado (PPD) e o teste anti-HIV foram negativos.

Apesar do aumento de provas inflamatórias e das imunoglobulinas totais, não foi possível definir uma etiologia (autoimune ou infecciosa). Na tentativa de diminuir a resposta inflamatória sistêmica e melhorar os resultados cirúrgicos, a reumatologia pediátrica indicou teste terapêutico com metilprednisolona (30 mg/kg/dia) por 5 dias. Previamente à pulsoterapia, foi realizada profilaxia com albendazol. Após o uso do corticoide, foram repetidos os exames, com a melhora do VHS (32 mm/h) e do PCR (< 5 mg/L). Dessa forma, para auxiliar no diagnóstico através de biópsia e cultura do aneurisma, bem como para evitar complicações como rotura, tromboembolia e dissecção, foi realizado o tratamento cirúrgico.

O acesso cirúrgico escolhido foi incisão em J na fossa ilíaca direita e dissecção do espaço extraperitoneal (incisão de Gibson). Após o reparo proximal da artéria ilíaca comum e distal da artéria ilíaca externa (Figura 2), foram realizadas abertura do aneurisma sacular e sutura hemostática do óstio da artéria ilíaca interna. Foi feita a remoção dos trombos e ressecção do segmento aneurismático; a reconstrução vascular foi uma anastomose término-terminal entre a artéria ilíaca comum e a artéria ilíaca externa, com pontos separados de polipropileno 6.0 (Figura 3) com auxílio de lupa cirúrgica com aumento 2,5 vezes. O procedimento transcorreu em 4 horas, sem intercorrências e sem necessidade de transfusão sanguínea. A análise anatomopatológica da parede do aneurisma e dos trombos foi de espessamento fibroso mural e intimal com ateroma focalmente calcificado, trombos recentes e em organização. A cultura foi negativa. O aneurisma foi classificado como congênito/idiopático.

**Figura 2:** Foto intraoperatória do aneurisma idiopático de artéria ilíaca comum direita isolado, com controle proximal e distal.

**Figura 3:** Foto intraoperatória mostrando o aspecto final da anastomose término-terminal das artérias ilíaca comum e ilíaca externa direita (seta) após a ressecção do aneurisma.

O paciente foi mantido em observação em unidade de terapia intensiva por 48 horas. Durante os primeiros 6 dias de pós-operatório, ficou internado e recebeu enoxaparina 10 mg/dia. No exame físico diário, constatou-se membros inferiores simétricos, bem perfundidos e pulsos periféricos também simétricos. A USG com Doppler demonstrou perviedade da anastomose. Após a alta hospitalar, o paciente permaneceu em uso de ácido acetilsalicílico 50 mg ao dia.

No seguimento ambulatorial, 1 mês após a cirurgia, o membro inferior direito estava sem edema, sem lesões cutâneas, mas com pulso periférico não palpável. Na USG com Doppler de controle, foi observada oclusão a nível da anastomose, porém com circulação colateral e sem repercussão clínica. Após 13 meses de seguimento com conduta expectante, não foi observada diferença de trofismo entre os membros.

## DISCUSSÃO

Os aneurismas são patologias raras em pediatria, principalmente de vasos de grande calibre e isolados^5^. Sarkar et al.^2^ classificaram os aneurismas arteriais em crianças em nove tipos: I) causado por infecção arterial; II) aortoarterite de célula gigante; III) doença autoimune do tecido conjuntivo; IV) doença de Kawasaki; V) com degeneração da camada média (síndromes de Marfan e Ehlers-Danlos); VI) associados a outras formas não inflamatórias de degeneração da camada média; VII) associados a displasias vasculares; VIII) aneurismas congênitos/idiopáticos; IX) pseudoaneurismas devido a lesão vascular.

Os aneurismas congênitos/idiopáticos são incomuns e exigem uma investigação detalhada para exclusão de outras causas^3^. No caso relatado, o aneurisma foi assim classificado devido à ausência de achados clínicos, laboratoriais ou anatomopatológicos que indicassem outras etiologias, como infecção, trauma, malformação vascular ou doenças do tecido conjuntivo.

Com base na história pregressa de amigdalites de repetição, a principal hipótese era de aneurisma micótico. Todavia, não foi confirmado na biópsia, e o paciente não teve novas infecções após o tratamento adequado. A pesquisa de autoanticorpos auxilia no diagnóstico pré-operatório de vasculites autoimunes. No exame físico, a avaliação da estatura, da hipermobilidade articular, da pele e das características de face e membros são fundamentais para a busca de alterações genéticas e autoimunes, guiando a investigação. Os antecedentes patológicos, a história familiar e o exame físico completo são cruciais para o diagnóstico correto. O achado histológico de fibroplasia da íntima é uma alteração frequente nos aneurismas idiopáticos, sendo, provavelmente, uma lesão secundária ao processo e não um fator etiológico^2^.

O tratamento cirúrgico é a indicação padrão por causa da evolução natural com rotura, trombose e tromboembolia^2^^,^ 3. Foram relatados na literatura o uso de enxertos (próteses, veias crio preservadas e enxertos autólogos), embolização, plicatura e ressecção com anastomose primária^1^^,^ 3 ^,^ 6 ^-^ 9. Essa última foi a abordagem de eleição. Recomenda-se utilizar pontos simples em toda a circunferência da anastomose, uma vez que a sutura contínua pode ser um fator de risco para estenose ao longo do crescimento da criança^6^^,^ 10. Há ainda a abordagem endovascular, a qual vem surgindo como uma alternativa promissora nas correções vasculares pediátricas^11^.

Os pacientes com aneurismas idiopáticos devem ser acompanhados devido ao risco de recidiva, novos aneurismas ou complicações da correção. A USG com Doppler é o exame de imagem de escolha^10^. Davis et al.^12^ recomendam o seguimento anual. Outra recomendação é uso de antiagregante plaquetário por 6 meses após a intervenção.

Neste paciente, foi mantida a antiagregação plaquetária e foi identificada oclusão ao nível da anastomose após 1 mês de seguimento, sem repercussões clínicas. Foi optada pela conduta expectante e não houve discrepâncias de trofismo entre os membros até a consulta mais recente, após 13 meses de seguimento. Todavia, é necessário seguimento a longo prazo para exclusão de sequelas relacionadas ao desenvolvimento da criança e do membro.

Conclui-se que o manejo de aneurismas de artéria ilíaca em crianças é um desafio tanto pela raridade dos casos quanto pelas limitações anatômicas (dimensões) e pelo crescimento da criança. A conduta cirúrgica é a de escolha, mas não há consenso sobre o momento exato de indicação e quanto à técnica cirúrgica preferencial. No caso relatado, optou-se pela ressecção e anastomose primária. Apesar da oclusão da anastomose após 1 mês, obteve-se sucesso no tratamento do aneurisma. Até o momento, não foram observadas sequelas que afetaram o desenvolvimento do paciente, sendo fundamental o seguimento a longo prazo.
